# Thin Film Flow in MHD Third Grade Fluid on a Vertical Belt with Temperature Dependent Viscosity

**DOI:** 10.1371/journal.pone.0097552

**Published:** 2014-06-20

**Authors:** Taza Gul, Saed Islam, Rehan Ali Shah, Ilyas Khan, Sharidan Shafie

**Affiliations:** 1 Department of mathematics, Abdul Wali Khan University Mardan, Mardan, KPK, Pakistan; 2 Department of mathematics, U.E.T Peshawar, Peshawar, KPK, Pakistan; 3 College of Engineering Majmaah University, Majmaah, Saudi Arabia; 4 Department of mathematical Sciences, Faculty of science, University Teknology Malaysia, UTM Johor Bahru, Johor, Malaysia; Texas A&M University, United States of America

## Abstract

In this work, we have carried out the influence of temperature dependent viscosity on thin film flow of a magnetohydrodynamic (MHD) third grade fluid past a vertical belt. The governing coupled non-linear differential equations with appropriate boundary conditions are solved analytically by using Adomian Decomposition Method (ADM). In order to make comparison, the governing problem has also been solved by using Optimal Homotopy Asymptotic Method (OHAM). The physical characteristics of the problem have been well discussed in graphs for several parameter of interest.

## Introduction

The subject of non-Newtonian fluids is popular and is an area of active research specially in mathematics, industry and engineering problems. Examples of non-Newtonian fluids include plastic manufacturing, performance of lubricants, food processing, movement of biological fluids, wire and fiber coating, paper production, transpiration cooling, gaseous diffusion, drilling mud, heat pipes etc. These fluids are described by a non-linear relationship between stress and the rate of deformation tensors and therefore several models have been proposed. There are several subclasses of non-Newtonian fluids. Third grade fluid is one of the important fluid in this category and its equation is based on strong theoretical foundations, where relation between stress and strain is not linear. Therefore, in this problem, we have considered third grade fluid. Considerable efforts have been made to study non-Newtonian fluids for various geometrical configurations via analytical techniques. Some developments in this direction are discussed in [1]–[19]. On the other hand, the physical importance of thin film has been highlighted by scientists and engineers. Amongst them, Khalid and Vafai [20] studied hydrodynomic squeezed flow and heat transfer over a sensor surface. Miladinova et al. [21] investigated thin film flow of a power law liquid falling from an inclined plate where it was observed that saturation of non-linear interaction occur in a permanent finite amplitude wave.

Similarly, Taza Gul et al. [22] investigated effects of slip condition on thin film flow of third grade fluids for lifting and drainage problem under the condition of constant viscosity. The effects of various parameters on the lift and drainage velocity profiles are also studied.

It is crystal clear that the physical problems are frequently modeled, using non-linear differential equations. Recently, several analytical and numerical techniques were used for solution of such non-linear problems. In order to find analytical approximate solutions of non-linear differential equations, researchers usually use approximate techniques such as Homotopy Perturbation Method (HPM) [23], Homotopy Analysis Method (HAM) [24] and Optimal Homotopy Asymptotic Method (OHAM) [25]. OHAM is a powerful mathematical technique and has already been applied to several non-linear problems. Marinca and Herisanu [26] used OHAM for solving non-linear equations arising in heat transfer problems. In another paper, Marinca [27] applied OHAM to study steady flow of a fourth grade fluid past a porous plate. Joneidi et al. [28] analyzed micropolar flow in a porous channel with high mass transfer. Siddiqui et al. [29] examined a thin film flow of non-Newtonian fluid over a moving belt. In another study, Siddiqui et al. [30] discussed the thin film flow of a fourth grade fluid down a vertical cylinder. Costa and Macedonio [31] noticed that increase in velocity may produce additional growth of local temperature. Nadeem and Awais [32] investigated thin film unsteady flow with variable viscosity. They analyzed the effect of variable thermo capillarity on the flow and heat transfer. Ellahi and Riaz [33] discussed analytical solution for MHD flow in a third grade fluid with variable viscosity. Whereas Aksoy et al. [34] found an approximate analytical solution for flow of a third grade fluid through a parallel plate channel filled with a porous medium.

The main objective of this research is to study thin film flow of MHD third grade fluid over a vertical belt under the influence of temperature with variable viscosity. More exactly, we are interested in showing the effects of MHD and variable viscosity with heat transfer in a thin film fluid flow such as silicate melts and polymers. In these fluids, viscous friction generates a local increase in temperature near the belt with decrease in resultant viscosity and frequently increases the flow velocity. The governing problem is solved using an analytical technique known as Adomian Decomposition Method (ADM). This technique was introduced by Adomian [35], [36] for finding the approximate solutions for linear and non-linear differential equations. Wazwaz [37], [38] used ADM for reliable treatment of Bratu-type and Rmden-Fowler equations. For comparisons and accuracy of results, the governing problem has also been solved by using OHAM.

## Basic Equations

The continuity, momentum and energy equations for incompressible, isothermal and electrically conducting third grade fluid are;
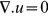
(1)




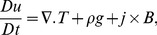
(2)

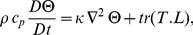
(3)


Here, 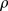 is the constant density, 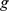 denotes gravitational acceleration, 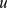 is the velocity vector of the fluid, 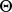 defines temperature, 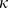 is the thermal conductivity, 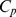 is specific heat, 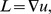 
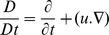
 denotes material time derivative, 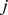 is the current density and 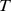 is the Cauchy stress tensor. Moreover, a uniform magnetic field 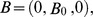 is applied in a direction, perpendicular to the belt. The Lorentz force per unit volume is given by
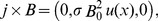
(4)


The Cauchy stress tensor 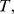 is given by
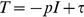
(5)where 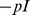 denotes spherical stress, 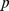 is the hydrostatic pressure and shear stress tensor 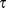, is defined as



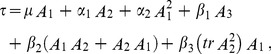
(6)Here 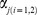 ,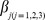 are the material constants and *A*_0,_ *A*_1,_ *A*_2_, *A*_3_ are the kinematical tensors given by
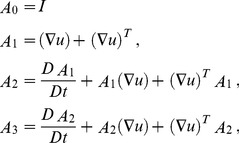
(7)


### Formulation of Lift Problem

Consider, a wide flat belt moves vertically upward at a constant speed *U* through a large bath of third grade liquid. The belt carries a layer of liquid of constant thickness, *δ* with itself. For analysis, coordinate system is chosen in which the y-axis is taken parallel to the surface of the belt and x-axis is perpendicular to the belt. Uniform magnetic field is applied transversely to the belt. It is assumed that the flow is steady and laminar after a small distance above the liquid surface layer and the external pressure is atmospheric everywhere.

Velocity and temperature fields are
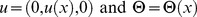
(8)


Using the velocity field given in Eq. (8) the continuity Eq. (1) satisfies identically and Eq. (5) gives the following components of stress tensor:
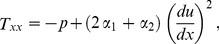
(9)




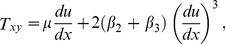
(10)




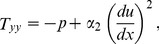
(11)




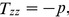
(12)




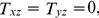
(13)


Incorporating Eqs. (9–13) into the momentum and energy equations (2, 3), we get
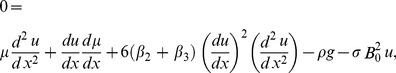
(14)




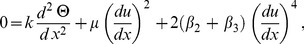
(15)


The corresponding boundary conditions are:
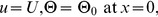
(16)




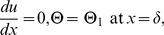
(17)


Introducing the following non-dimensional variables
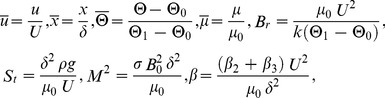
(18)where *B_r_* is the Brinkman number , *M* ^2^ is the magnetic parameter, 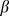 is the non-Newtonian parameter and *S_t_* is the Stock’s number.

For Reynold’s model, the dimensionless viscosity
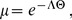
(19)


Using Taylor series expansion, one may represent viscosity and its derivative as follows:
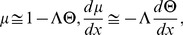
(20)


Using the above dimensionless variables into Eqs. (14–17) and dropping out the bar notations, we obtain.

Eq. (14) has been rectified for Eq. (21, 22)
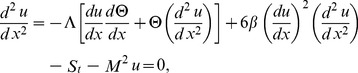
(21)




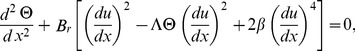
(22)




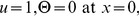
(23)




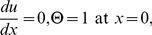
(24)


### Solution of Lifting Problem

#### The OHAM solution

In order to solve the system of equations (21–24), we define the linear, non-linear functions and source terms respectively as follows:
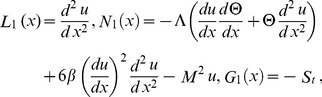
(25)




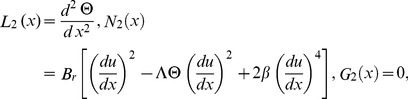
(26)


Now, OHAM is applied to non-linear coupled ordinary differential Eqs. (21, 22) and Eqs. (25, 26) as follows:
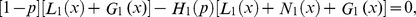


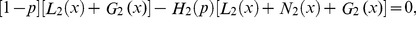
(27)


We consider 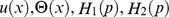 as the following.
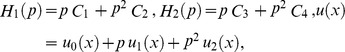


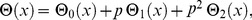
(28)


Substituting 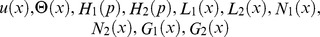 from Eq. (28) into Eq. (27) and after some simplifications based on power of p-terms, we get the following.

Zero components:
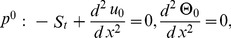
(29)


First components:
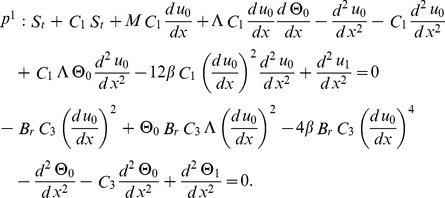
(30)


Solving Eqs. (29, 30) along with boundary conditions (23, 24), we get the term solutions as fallows.

Zero term solution:
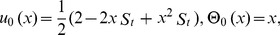
(31)


First term solution:
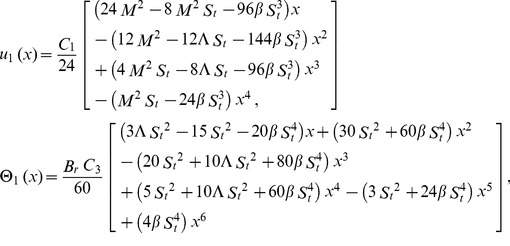
(32)


The second term solution for velocity and temperature are too bulky, therefore, only graphical representations up to second order are given.

The series solutions of velocity profile and temperature distribution are
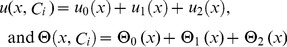
(33)


The arbitrary constants 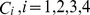 are found out by using the residual
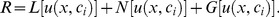
(34)


For velocity profile and temperature distribution the arbitrary constants are mentioned in graphs.

The constants 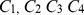 can also be obtained from Collocation and Ritz methods.

#### The ADM solution

The inverse operator 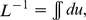 of the ADM on the second order coupled Eqs. (21,22) is used:
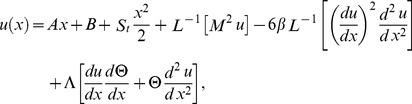
(35)




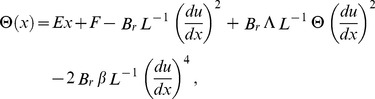
(36)


The series solutions of Eqs. (35, 36):
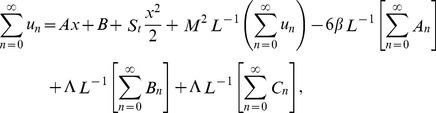
(37)




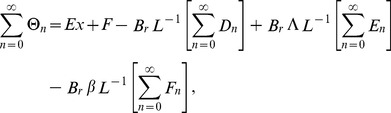
(38)


The Adomian polynomials 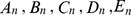 and 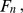 for Eqs. (37, 38) are defined as
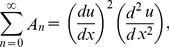
(39)




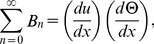
(40)




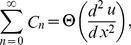
(41)




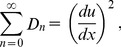
(42)




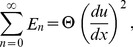
(43)




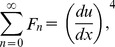
(44)


The components of Adomian polynomials are derived from Eqs. (39–44) as:
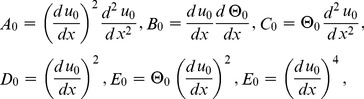
(45)

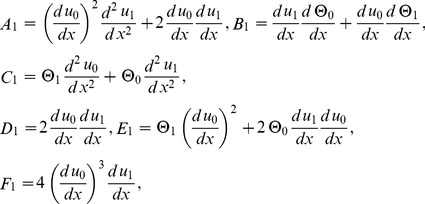
(46)


The series solutions of Eqs. (37, 38) are derived as:
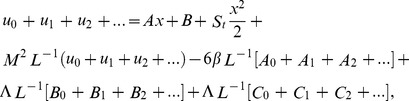
(47)

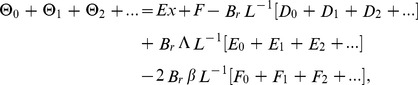
(48)


The velocity and temperature components are obtained by comparing both sides of Eqs. (47, 48):

Components of the lift problem up to second order are:
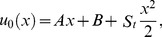
(49)




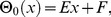
(50)




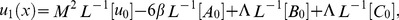
(51)




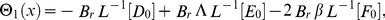
(52)




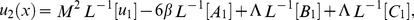
(53)




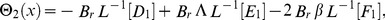
(54)subject to the boundary conditions



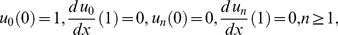
(55)

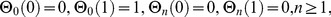
(56)


Using boundary conditions from Eqs. (55, 56) into Eqs. (49–54), we obtain
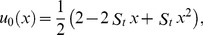
(57)




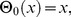
(58)




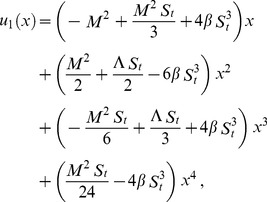
(59)

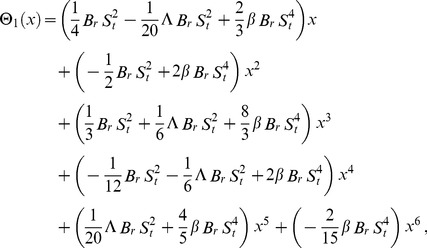
(60)


Due to lengthy calculations, the analytical results have been given up to first order but they have been shown graphically up to second order.

### Formulation of Drainage Problem

Under the same assumptions as in the previous problem, we consider a film of non-Newtonian liquid draining down the vertical belt. The belt is stationary and the fluid drains down the belt due to gravity. The gravity in this case is opposite to the previous case. The coordinate system is selected same as in the previous case. Assuming that the flow is steady and laminar, external pressure is neglected whereas the fluid shear forces keep gravity balanced and the thickness of the film remains constant.

Boundary conditions for the drainage problem are
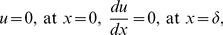
(61)


Using non-dimensional variables, the boundary conditions for drainage problem become
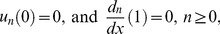
(62)


For temperature distribution, the boundary conditions are same as given in Eq. (56).

### Solution of Drainage Problem

#### The OHAM solution

From Eqs. (21, 22), the linear, non-linear functions and source term (in drainage case), it is opposite due to gravity) are respectively defined as
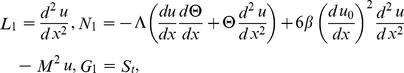
(63)

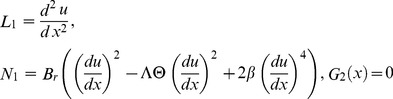
(64)


OHAM is applied to non-linear coupled ordinary differential Eqs. (63, 64) as
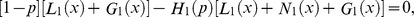


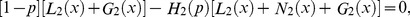
(65)


We consider 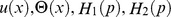 as the following
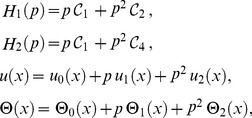
(66)


Substituting 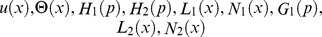 and 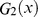 from Eq. (66) into Eq. (65) we have the following components of velocity and temperature.

Zero components:
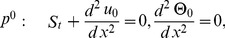
(67)


First components:
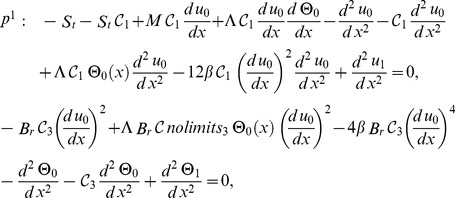
(68)


Solving Eqs. (67, 68) with boundary conditions (61, 62), we get
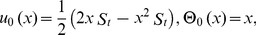
(69)

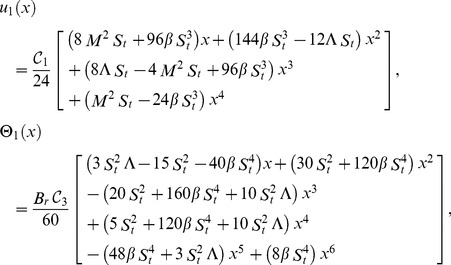
(70)


Like previous problem, results up to first order terms haven been obtained.

#### ADM solution

Using ADM on Eqs. (21, 22), the Adomian polynomials in equations (45, 46) for both problems are same whereas the different velocity components are obtained as:

### Components of the Problem

The boundary conditions of first and second components for drainage velocity profile are same as given in Eq. (56). Also, the boundary conditions for temperature distribution are same as given in Eq. (57) but solution of these components is different, depends on the different velocity profile of drainage and lift problems. Due to lengthy analytical calculation, solutions up to first order terms are included whereas the graphical representations up to second order terms are given. Using boundary conditions (62) and (56) into Eqs. (49–54), the components, solution are obtained as:
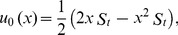
(71)




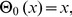
(72)




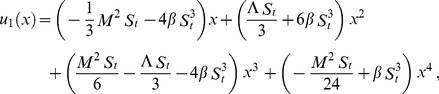
(73)




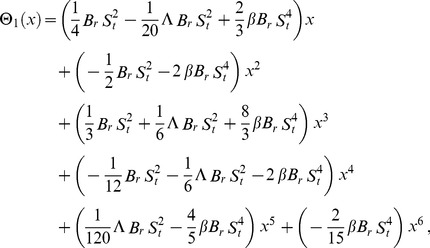
(74)


## Results and Discussion

**Figure 1:**
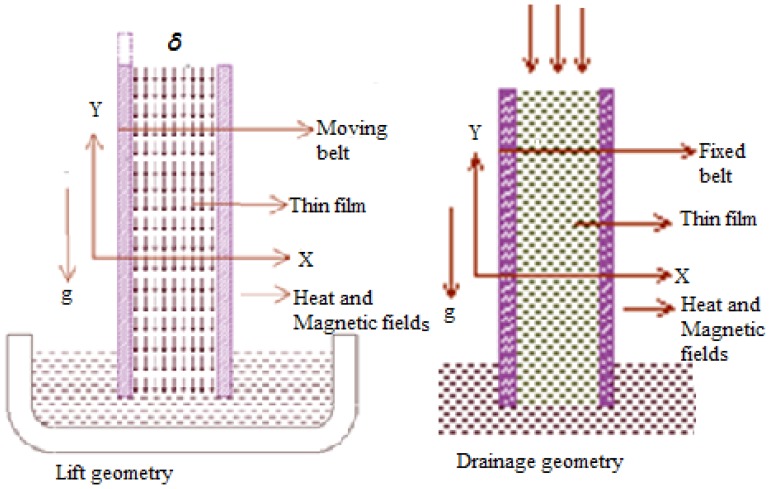
Geometry of the problem (a) Lift problem and (b) Drainage problem.

**Figure 2:**
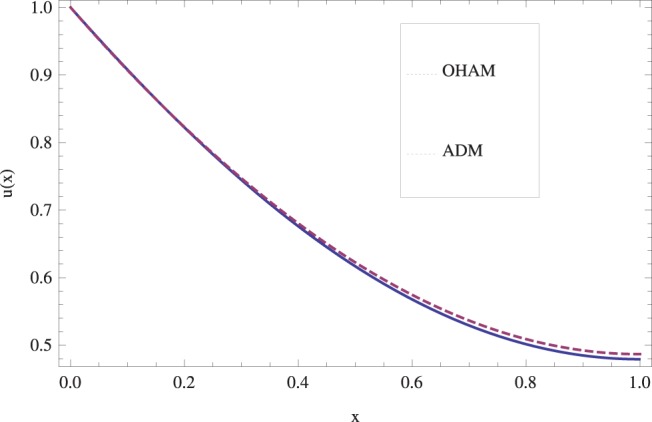
Comparison of ADM and OHAM methods for lift velocity profile. 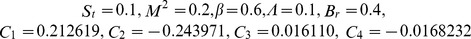.

**Figure 3:**
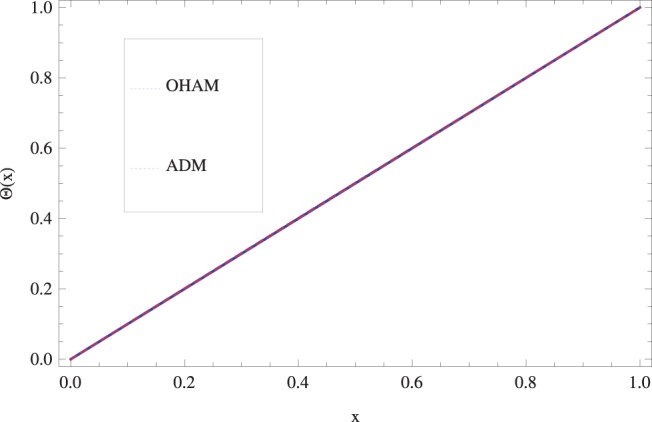
Comparison of ADM and OHAM methods for lift temperature distribution.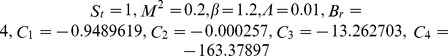.

**Figure 4:**
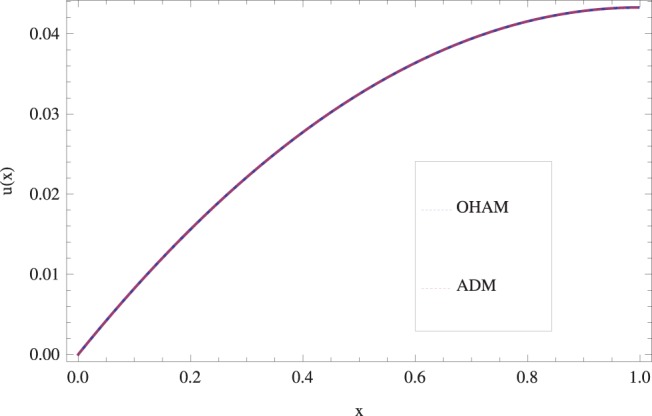
Comparison of ADM and OHAM methods for drainage velocity profile.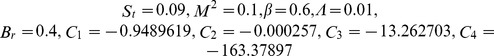.

**Figure 5:**
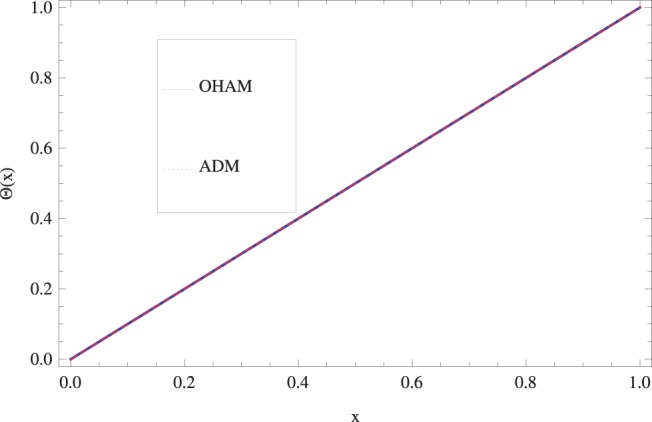
Comparison of ADM and OHAM methods for drainage temperature distribution.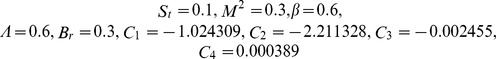.

**Figure 6:**
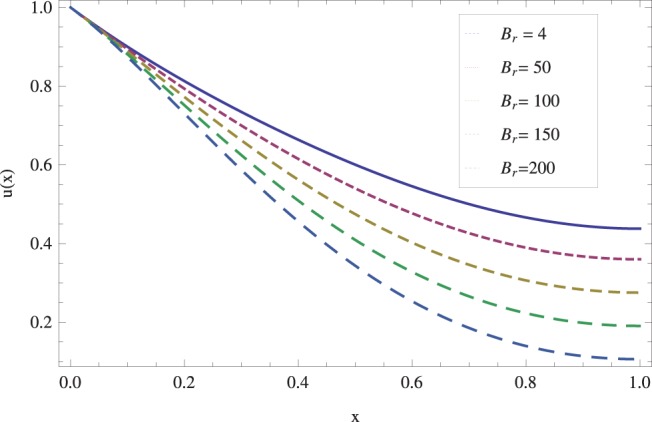
Influence of the Brinkman number on the lift velocity profile when 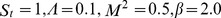
_._

**Figure 7:**
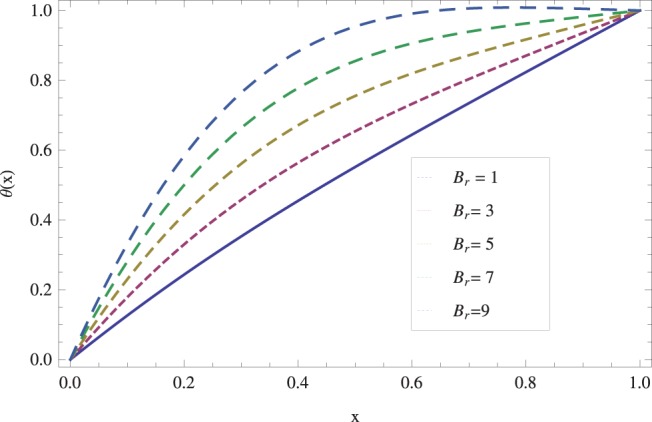
Influence of the Brinkman number on the lift temperature distribution when 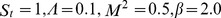
_._

**Figure 8:**
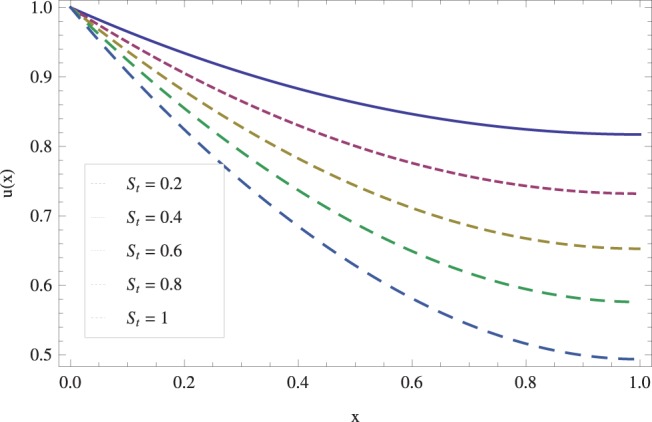
Effect of Stock number on the lift velocity profile. when 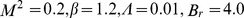
_._

**Figure 9:**
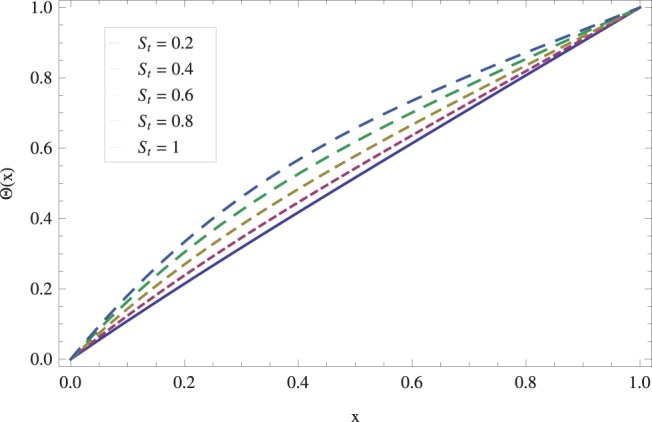
Effect of Stock number on the lift temperature distribution when 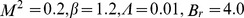
_._

**Figure 10:**
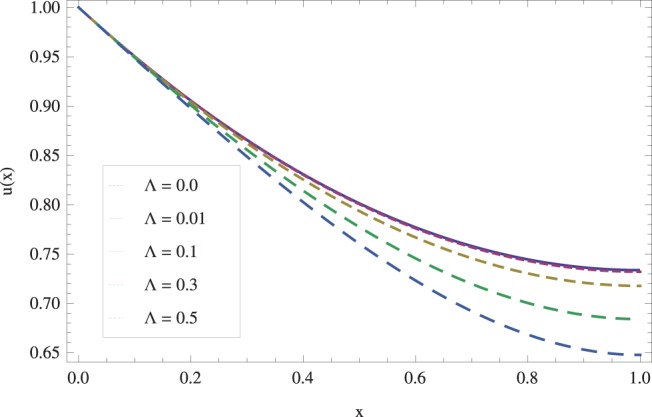
Effect of viscosity parameter on the lift velocity profile. when 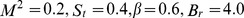
_._

**Figure 11:**
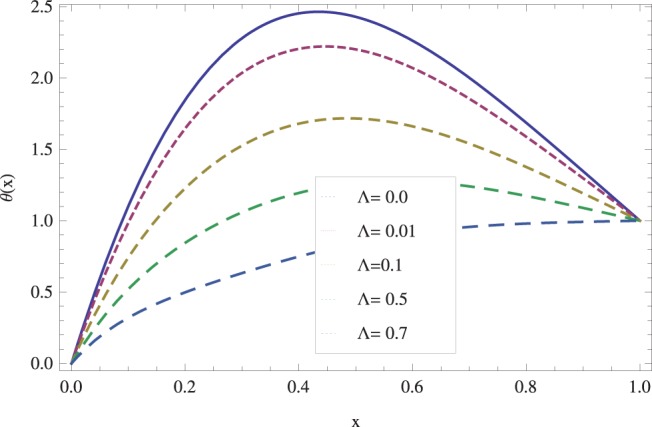
Effect of viscosity parameter on the lift temperature distribution when 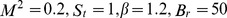
_._

**Figure 12:**
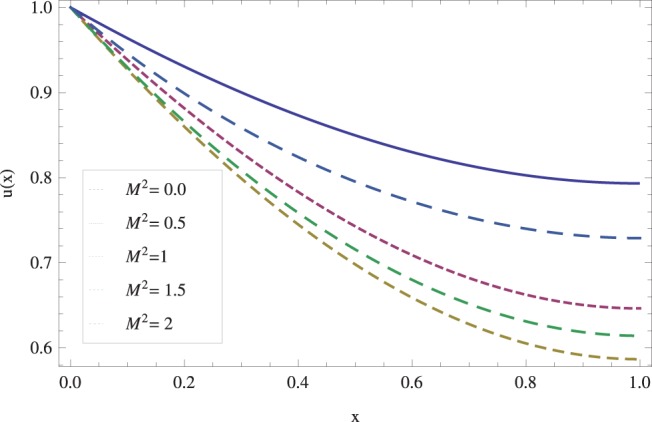
The effect of magnetic force on lift velocity profile when 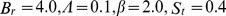
_._

**Figure 13:**
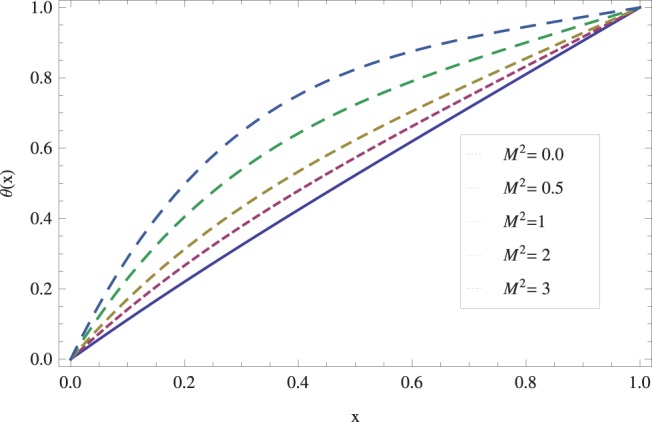
The effect of magnetic force on lift temperature distribution when 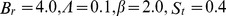
_._

**Figure 14:**
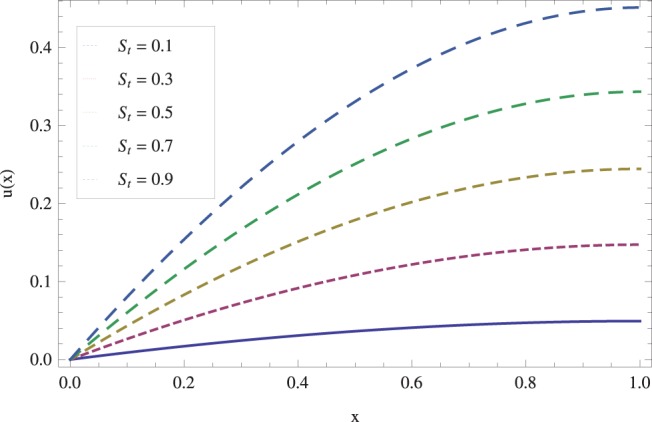
The effect of Stock number on velocity for drainage problem when 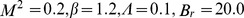
_._

**Figure 15:**
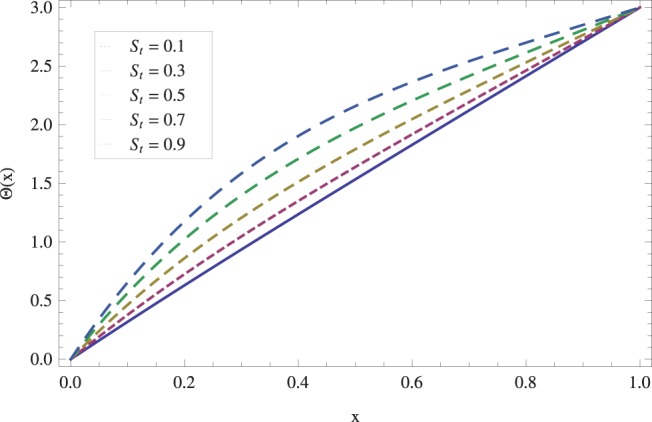
The effect of Stock number on temperature for drainage problem when 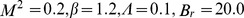
_._

**Figure 16:**
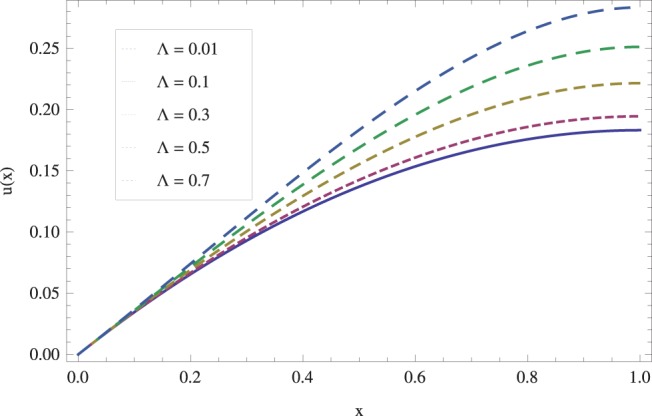
Drain velocity for various values of viscosity parameter when 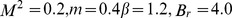
_._

**Figure 17:**
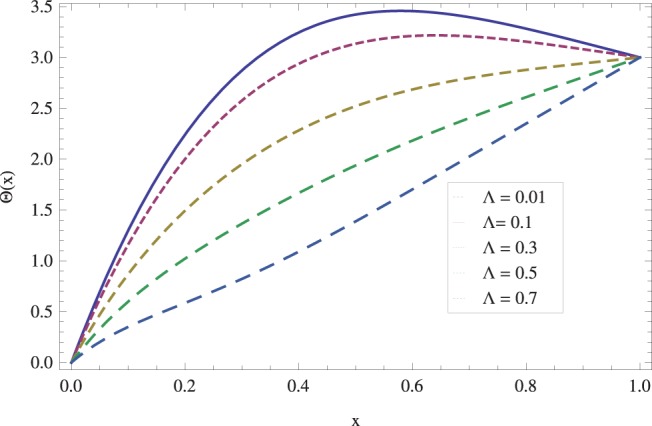
Drain temperature distribution for various values of viscosity parameter when 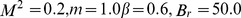
_._

**Figure 18:**
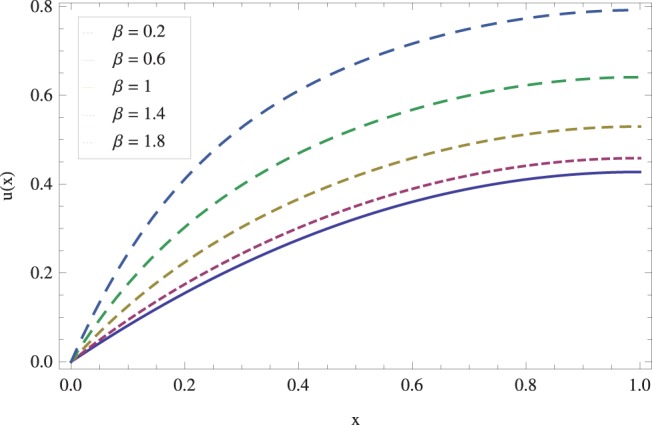
The influence of non-Newtonian parameter 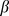 on velocity for drainage problem when 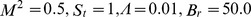
_._

**Figure 19:**
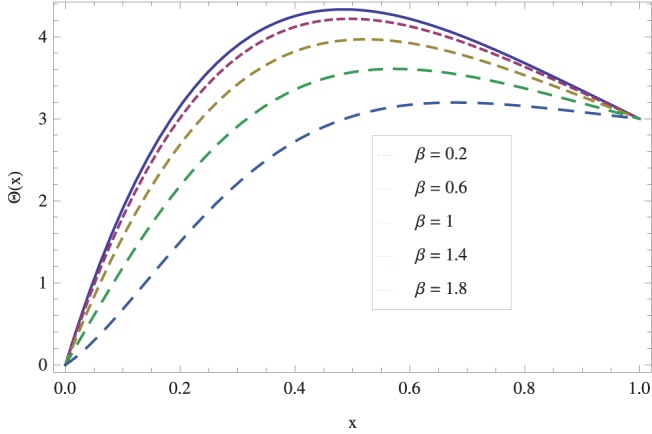
The influence of non-Newtonian parameter 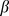 on temperature for drainage problem when 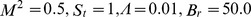
_._

**Figure 20:**
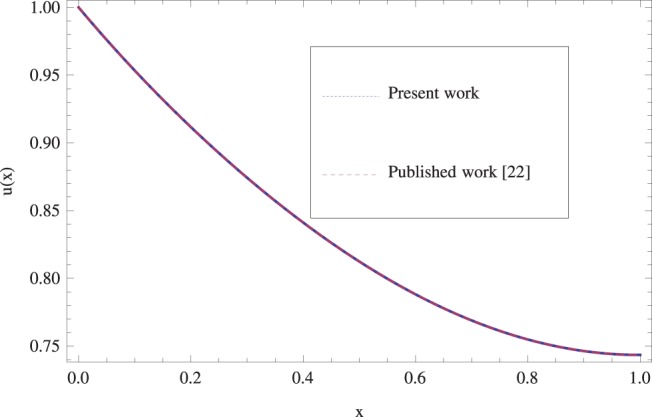
Comparison of the present results with published work [22] when 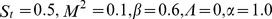.

The effect of Stock number 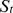 magnetic parameter 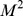, Brinkman number 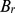 non-Newtonian parameter 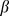 and viscosity parameter 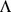 in lifting and drainage problems together with the physical interpretation of the problem have been discussed in Figs. 1–20. Fig. 1 shows the geometry of lift and drainage problems. A comparison of the ADM and OHAM solutions is shown in Figs. 2–5 for various values of physical parameters. It is found from these figures that ADM and OHAM solutions are in good agreement. Figs. 6 and 7 provide variation of velocity and temperature distribution for different values of Brinkman number. It has been found that velocity decreases whereas temperature inside the fluid increases by increasing 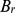 while keeping the other parameters fixed. In Fig. 8, we observed that velocity decreases with an increase in the Stock number 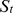. Physically, it is true as increasing Stock number causes the fluid^’^s thickness and reduces its flow. The effect of Stock number 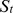 on temperature distribution has been illustrated in Fig. 9. It is observed that temperature *Θ* increases monotonically for large values of Stock number 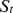. The effect of viscosity parameter 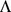 on lift velocity u is shown in Fig. 10. It is observed that the speed of flow decreases by increasing 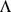. The speed of flow is actually caused by shear^’^s thickening and thinning effects due to increase and decrease in viscosity parameter. A similar situation is observed in Fig. 11 where an increase in viscosity parameter 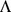 decreases temperature distribution. Here, the velocity profiles are parabolic in nature and their amplitudes depend on the magnitude of the viscosity parameter 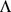. Variations of the magnetic parameter 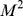 on lift velocity have been studied in Fig. 12. Here, it is clear that the boundary layer thickness is reciprocal to the transverse magnetic field and velocity decreases as flow progresses towards the surface of the fluid. On the other hand, temperature profile as shown in Fig. 13 indicates that fluid temperature increases with magnetic parameter. Fig. 14 shows that velocity increases in drainage flow when Stock number 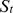 increases. Physically, it is due to friction which seems smaller near the belt and higher at the surface of the fluid. Further, it is found from Fig. 15 that temperature profile also increases when 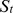 is increased. Fig. 16 illustrates the effect of variable viscosity parameter 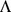 on the drain flow. It is observed that at higher values of viscosity parameter 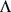, velocity of the fluid increases gradually towards the surface of the fluid. However, it is found from Fig.17, that an increase in viscosity parameter 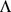 causes gradual decrease in temperature field. The effects of non-Newtonian parameter 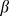 on drain velocity have been studied in Fig. 18. We observed that an increase in 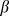 raises drain velocity profile and decreases temperature profile as shown in Fig. 19. Finally for the accuracy purpose the present results are compared with published work in [22] in Fig. 20 and in table 1. An excellent agreement is found.

**Table 1:** Numerical results show the comparison of present results with published work [22] when 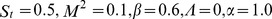.

x	Present work	Published work[22]	Absolute error
**0**	1	1	0
**0.1**	0.95336886435638888	0.95336886432889	1.11022×10^−16^
**0.2**	0.91163368	0.91163368	1.11023×10^−16^
**0.3**	0.8742813014374999	0.8742813014375	0
**0.4**	0.84116332088888	0.84116332088888	0
**0.5**	0.8123818359375	0.8123818359375	0
**0.6**	0.788190612	0.788190612	0
**0.7**	0.76891138782638	0.76891138782638	0
**0.8**	0.754865408	0.754865408	0
**0.9**	0.74632017043749	0.7463201704375	0
**1.0**	0.7434513888888	0.7434513888888	0

### Future Work

We intend to carry out researches in future on third grade fluid on vertical belt regarding the following discussions:

1. Time dependent third grade fluid on vertical belt.
2. Vogel Model third grade fluid on vertical belt with slip boundary conditions.
3. Third grade fluid on vertical belt wit surface topography.
4. Third grade fluid on vertical rotating disc with surface topography.

## Conclusion

In this work, we have investigated the thin film flow non-Newtonian third grade fluid due to vertical belt and the fluid was subjected to lifting and drainage. Analytical solutions of the lifting and drainage problems have been obtained using ADM and OHAM. It has been shown graphically that these solutions are identical. The results for velocity and temperature have been plotted graphically and discussed in detail. It has been observed that these solutions are valid not only for small but also for large values of the emerging parameters. It has been observed that in both cases of lift problem velocity decreases while temperature increases with increasing Brinkman number 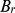. However, in drainage problem both velocity and temperature increases.
