# Correction: Thin Film Flow in MHD Third Grade Fluid on a Vertical Belt with Temperature Dependent Viscosity

**DOI:** 10.1371/journal.pone.0110205

**Published:** 2014-10-03

**Authors:** The PLOS ONE Staff

The affiliations for the fourth and fifth authors are incorrect. Ilyas Khan is not affiliated with #4 but with #3 College of Engineering Majmaah University, Majmaah, Saudi Arabia. Sharidan Shafie is not affiliated with #4 but with #4 Department of mathematical Sciences, Faculty of science, University Teknology Malaysia, UTM Johor Bahru, Johor, Malaysia.
